# Distinct Transcriptomic Profile Underlying High CO_2_ and Ethylene-Induced Deastringency in ‘Daebong’ Persimmon Fruit

**DOI:** 10.3390/cimb47090689

**Published:** 2025-08-26

**Authors:** Min Woo Baek, Se Min Chang, DoSu Park, Shimeles Tilahun, Cheon Soon Jeong

**Affiliations:** 1 Department of Horticulture, Kangwon National University, Chuncheon 24341, Republic of Korea; minwoo100@kangwon.ac.kr (M.W.B.); semin9933@kangwon.ac.kr (S.M.C.); parkds@kangwon.ac.kr (D.P.); 2 Interdisciplinary Program in Smart Agriculture, Kangwon National University, Chuncheon 24341, Republic of Korea; 3 Agriculture and Life Science Research Institute, Kangwon National University, Chuncheon 24341, Republic of Korea; 4 Department of Horticulture and Plant Sciences, Jimma University, Jimma 378, Ethiopia

**Keywords:** deastringency, ethylene, high CO_2_, soluble tannins, firmness

## Abstract

Astringent persimmons (*Diospyros kaki* Thunb.) require effective postharvest deastringency treatments due to their high soluble tannin content at harvest. While high CO_2_ and ethylene are commonly used to remove astringency, their different effects on fruit firmness and quality require cultivar-specific approaches. This study investigated the transcriptomic and biochemical responses of ‘Daebong’ persimmon to high CO_2_ and ethylene treatments during deastringency. Both treatments significantly decreased soluble tannin and total phenolic content, enhancing fruit edibility. However, the firmness was maintained under high levels of CO_2_, but it decreased quickly after exposure to ethylene. RNA-Seq analysis identified 2271 differentially expressed genes (DEGs) and revealed distinct transcriptional signatures for each treatment. CO_2_ treatment activated hypoxia-responsive genes, stress-related transcription factors (e.g., *WRKY*, *ERF14/26*), and components of antioxidant defense (e.g., *GSTU17*, *peroxidases*), which contributed to oxidative stress reduction and preservation of firmness. On the other hand, ethylene treatment increased ethylene biosynthesis (*ACS*), signaling (*EIN3-binding F-box*), and ripening-related genes (*polygalacturonase*, *laccase*, *ERF061/113*), which promote cell wall degradation and softening. Functional enrichment analysis revealed that various regulatory mechanisms are responsible for the insolubilization of tannins, loss of antioxidants, and changes in firmness. These findings provide new insights into the molecular responses of pollination-constant astringent (PCA) persimmons, particularly the ‘Daebong’ cultivar, to postharvest deastringency treatments for the development of more effective postharvest management strategies. The results suggest that high CO_2_ helps maintain fruit quality by promoting stress adaptation and suppressing pathways that lead to softening, whereas ethylene accelerates the ripening process by activating signaling pathways associated with ethylene.

## 1. Introduction

Persimmon (*Diospyros kaki* Thunb.) is a climacteric fruit widely cultivated in East Asia, particularly in China, Japan, and South Korea, which together account for more than 94.4% of global production [1]. Among the diverse astringent cultivars, ‘Daebong’, a traditional Korean cultivar classified as pollination-constant astringent (PCA), is highly valued for its rich flavor and is widely used for both fresh consumption and processing [2,3,4]. Unlike non-astringent cultivars, such as pollination-constant non-astringent (PCNA) types, PCA-type persimmons maintain high levels of soluble tannin at harvest. This results in a pronounced astringent taste that limits their direct edibility [5,6].

During the development of fruit, oriental persimmons accumulate water-soluble tannins in the tannin cells of their flesh, which are responsible for the characteristic astringent taste [5] These compounds interact with salivary proteins, resulting in an unpleasant drying or puckering sensation in the mouth [7]. Therefore, postharvest deastringency treatment is essential for the commercial handling and consumer acceptance of astringent cultivars like ‘Daebong’ [2]. Traditional treatments such as ethanol exposure [8], hot water immersion [9], and natural ripening have been reported to induce acetaldehyde accumulation, which polymerizes soluble tannins into insoluble forms [6]. However, these methods often lead to inconsistent quality and require extended treatment durations. Among the currently available methods, high CO_2_ and exogenous ethylene treatments are commonly used for their rapid and reliable deastringency effects [3,10]. High CO_2_ treatment creates anaerobic conditions, activating the pyruvate decarboxylase (PDC) and alcohol dehydrogenase (ADH) pathways, which leads to the accumulation of acetaldehyde [11,12,13]. In contrast, ethylene treatment enhances deastringency by regulating hormones and activating enzymes associated with ripening [14,15,16]. However, the two treatments have different effects on fruit firmness and postharvest handling. High CO_2_ treatment is generally effective in preserving flesh firmness and reducing astringency. In contrast, exposure to ethylene tends to cause rapid softening of the fruit by increasing the content of water-soluble pectin and promoting the degradation of cell walls [2,17,18].

Previous transcriptomic studies on deastringency have predominantly concentrated on Japanese PCNA cultivars [19] and Chinese PCA cultivars [20]. However, information regarding traditional Korean PCA-type cultivars, such as ‘Daebong’-a widely cultivated astringent persimmon in South Korea-remains relatively limited. Understanding the unique molecular and biochemical responses of ‘Daebong’ to high CO_2_ and ethylene is essential for optimizing postharvest treatments, given the commercial importance of firmness retention and consumer-preferred soft textures.

Therefore, this study aimed to investigate the specific transcriptomic and biochemical responses of the ‘Daebong’ persimmon cultivar to high CO_2_ and ethylene treatments. The research focused on identifying significant changes in gene expression, firmness characteristics, and shifts in antioxidant-related metabolites that occur during the deastringency process. The findings will contribute to the development of more effective postharvest management strategies for PCA-type persimmons.

## 2. Materials and Methods

### 2.1. Plant Materials, High CO_2_ and Ethylene Treatments

Mature ‘Daebong’ astringent persimmons (*Diospyros kaki* Thunb.) were harvested on 21 October 2023, from an orchard in Yeongam, Jeollanam-do, South Korea (latitude 34.84698° N, longitude 126.77564° E). Only fruits that were uniform in size and free from visible defects or external damage were selected for subsequent treatment. Immediately after harvest, the fruits were transported to the Postharvest Management Laboratory at the Department of Horticulture Science, Kangwon National University. At harvest, fruits of the ‘Daebong’ cultivar had an average fresh weight of 300 g, firmness of 21.31 N, and soluble tannin content of 5.52 g kg^−1^. For the deastringency treatments, 20 selected fruits were placed in 62 L airtight plastic chambers without any packaging to ensure uniform gas exposure. For the high CO_2_ treatment, 95% CO_2_ (purity ≥ 99.9%) was introduced into the chambers and maintained for 24 h at 25 °C, based on the total chamber volume (62 L) [21,22]. For the ethylene treatment, 100 μL L^−1^ ethylene (purity ≥ 99.9%) was applied under the same chamber and temperature conditions for 24 h [2,18]. Internal air circulation within the chambers was maintained using a small fan (EC12025L24S, Evercool Thermal Ltd., Dongguan, China) to ensure uniform gas distribution. Control fruits were held under identical chamber conditions (25 °C, 24 h) without high CO_2_ or ethylene treatments. Tissue samples were collected at three different time points: before the treatment began (0 days), after 1 day of high CO_2_ treatment, and after 4 days of ethylene treatment. These samples were used for transcriptome profiling, measuring pectin content, ethanol-insoluble solids (EIS), and polygalacturonase (PG) activity. The collected fruit flesh was immediately frozen in liquid nitrogen and stored at −80 °C until analysis. Simultaneously, fruit firmness, ethylene production, and respiration rates were measured using intact fruits. For the biochemical analyses of secondary metabolites and antioxidant activity, three biological replicates from each treatment group were also stored at −80 °C and subsequently freeze-dried using an FDT-8650 vacuum freeze dryer (Operon, Gimpo, Republic of Korea). The dried tissues were ground into a powder using a food grinder, filtered through a 40 μm mesh sieve, sealed in low-density polyethylene (LDPE) pouches, and preserved at −20 °C until extraction for analysis.

### 2.2. RNA Extraction

The overall transcriptome analysis followed the protocol described by [23]. Total RNA was extracted using the hybrid-RTM kit (Gene All Biotechnology Co., Seoul, Republic of Korea). RNA quality and quantity were assessed using the Agilent 2100 Bioanalyzer (Agilent Technologies Inc., Santa Clara, CA, USA). Only RNA samples with concentrations ranging from 1 to 10 µg and an RNA Integrity Number (RIN) above 8.0 were selected for downstream applications. Equal amounts of high-quality total RNA from each sampling time point were pooled to construct three RNA-Seq libraries corresponding to the untreated control, high CO_2_, and ethylene treatment groups.

### 2.3. cDNA Library Construction, Sequencing, and Quality Control

cDNA libraries were prepared using the TruSeq RNA Sample Preparation Kit v2 (Illumina Inc., San Diego, CA, USA) according to the manufacturer’s instructions. Paired-end sequencing was performed on an Illumina NovaSeq 6000 platform at Macrogen Inc. (Seoul, Republic of Korea). Raw reads were processed using Trimmomatic v0.39 (http://www.usadellab.org/cms/?page=trimmomatic (accessed on 4 December 2023)) with the following parameters: removal of adapter sequences, trimming of low-quality bases with Phred scores below 20, and exclusion of reads shorter than 50 bp. Raw reads were processed using Trimmomatic v0.39 (http://www.usadellab.org/cms/?page=trimmomatic (accessed on 4 December 2023)) with the following parameters: removal of adapter sequences, trimming of low-quality bases with Phred scores below 20, and exclusion of reads shorter than 50 bp [24].

### 2.4. De Novo Assembly and Contig Filtering

Sequencing reads were de novo assembled into contigs using the Trinity v2.13.2 with default parameters (https://github.com/trinityrnaseq/trinityrnaseq/wiki (accessed on 4 December 2023)). Redundant contigs were removed using CD-HIT-EST v4.8.1 with a sequence identity threshold of 0.9, retaining only the longest sequence among highly similar contigs (https://github.com/weizhongli/cdhit/releases (accessed on 4 December 2023)). The quality of the assembled transcriptome was evaluated by mapping clean reads back to the contigs using BWA v0.7.17 (https://github.com/lh3/bwa (accessed on 4 December 2023)) and SamTools v1.13 (https://www.htslib.org (accessed on 4 December 2023)). Contigs with fewer than five mapped reads were excluded from downstream analyses [25].

### 2.5. Identification of DEGs and Functional Enrichment Analysis

To identify coding sequences within the contigs, TransDecoder v5.5.0 (https://github.com/TransDecoder/TransDecoder/wiki (accessed on 4 December 2023)) was used. The TransDecoder.LongOrfs module was applied to predict the longest open reading frames (ORFs), which were then annotated by homology searches against the UniProt database using DIAMOND v2.1.8.162 and the Pfam database using HMMER v3.3 (http://hmmer.org/ (accessed on 4 December 2023)). The final coding sequence set was defined using the TransDecoder.Predict module. Redundant sequences were filtered using CD-HIT v4.8.1 at 95% identity, based on predicted protein sequences.

For expression analysis, clean reads were aligned to the assembled contigs using BWA v0.7.17, and read counts were generated using Samtools v1.13. Differentially expressed genes (DEGs) were identified using DESeq2 v1.36.0 with an adjusted *p*-value (FDR) < 0.05 and |log_2_ fold change| > 2 as thresholds to determine significant expression changes among the three treatment groups.

Functional annotation of the predicted proteins was conducted using DIAMOND v2.1.8.162 against the NCBI non-redundant (nr) database. InterProScan v5.62-94.0 (https://www.ebi.ac.uk/interpro/search/sequence/ (accessed on 4 December 2023)) was also employed to identify conserved protein domains. All annotation results were integrated using BLAST2GO (https://www.blast2go.com/ (accessed on 4 December 2023) to assign Gene Ontology (GO) terms and to perform enrichment analysis. Transcriptome completeness was evaluated using BUSCO v5.3.2 with the eukaryota lineage dataset, providing a standardized measure of assembly quality based on the detection of conserved single-copy orthologs.

### 2.6. Firmness, Ethanol Insoluble (EIS), Pectin Content, and Polygalacturonase (PG) Activity

Fruit firmness was measured using a hardness meter (Rheometer, Sun Scientific Co., Ltd., Tokyo, Japan) fitted with a cylindrical stainless-steel probe, measuring 3 mm in diameter and having a flat end. Measurements were taken at the equatorial region of 15 randomly selected fruits, with two replicates per fruit. A maximum compression force of 98.1 N (equivalent to 10 kgf) was applied. The firmness values were recorded as the peak force required penetrating the surface of the fruit, following the methodology described by Baek et al. [2]. Ethanol-insoluble solids (EIS), total pectin content, and polygalacturonase (PG) activity were measured using established protocols with slight modifications, as described by Choi et al. [26] and Seo et al. [27]. EIS was extracted by homogenizing the fruit tissue in ethanol and recovering the insoluble fraction through centrifugation. Total pectin content was quantified using colorimetric analysis following acid extraction, while PG activity was evaluated by measuring the rate of hydrolysis of polygalacturonic acid under specific pH and temperature conditions.

### 2.7. Ethylene Production and Respiration Rate

The ethylene production and respiration rates of persimmon fruits were determined following the procedure described by Choi et al. [26], with slight modifications to the gas chromatography conditions according to the actual instrument settings. Intact fruits were sealed in 4 L airtight containers and incubated for 3 h at an ambient temperature. A 1 mL headspace gas sample was withdrawn from each container using a gas-tight syringe and injected into a gas chromatograph (Nexis GC-2030, Shimadzu Corporation, Kyoto, Japan) equipped with a BP20 fused silica capillary column (30 m × 0.25 mm i.d., 0.25 μm film thickness; SGE Analytical Science, Victoria, Australia) and a flame ionization detector (FID). The injector (SPL) and detector (FID) temperatures were both maintained at 250 °C. The column oven was set to 160 °C under isothermal conditions. Helium was used as the carrier gas at a constant total flow rate of 15.0 mL min^−1^ with a split ratio of 5.0. The make-up gas (He) flow was maintained at 24.0 mL min^−1^, while hydrogen and air flows for the FID were set to 32.0 mL min^−1^ and 200.0 mL min^−1^, respectively. The column pressure was maintained at approximately 195.7 kPa. The linear velocity was 46.6 cm s^−1^ and the purge flow was 3.0 mL min^−1^. The rate of ethylene production was expressed as microliters of C_2_H_4_ per kilogram of fruit per hour (μL C_2_H_4_ kg^−1^ h^−1^).

For respiration analysis, CO_2_ accumulation in the headspace was quantified using a headspace gas analyzer (GS 6600, Illinois Instruments, Inc., Johnsburg, IL, USA). CO_2_ concentration was measured at the beginning and after 3 h of incubation, and the respiration rate was calculated based on the increase in CO_2_ concentration over time. Results were expressed as milliliters of CO_2_ per kilogram per hour (mL CO_2_ kg^−1^ h^−1^).

### 2.8. Soluble Tannin Content and Total Phenolics

Soluble tannin was extracted from freeze-dried persimmon powder using 80% aqueous methanol by applying two sequential extraction steps: ultrasonication and orbital shaking (SI-600R, Medline Scientific, Oxfordshire, UK), each for 1 h at room temperature, followed the method described by Tewari et al. [28] (2017). The resulting extracts were centrifuged at 12,578× *g* for 15 min at 22 °C using a Mega-17R refrigerated centrifuge (Hanil Science Industrial, Gimpo, Republic of Korea). The supernatant was filtered through a 0.22 μm PTFE syringe filter and transferred into HPLC vials for chromatographic analysis.

Chromatographic separation was conducted using a high-performance liquid chromatography system (Nanospace SI-2, Shiseido Co., Kyoto, Japan) equipped with a photodiode array detector (PDA). A reversed-phase Cadenza 5CD-C18 column (4.6 × 250 mm, 5 μm; Imtakt Co., Kyoto, Japan) was employed, and column temperature was maintained at 40 °C. The detection wavelength was set at 270 nm. The mobile phase consisted of 0.1% (*v*/*v*) trifluoroacetic acid in distilled water (solvent A) and methanol (solvent B). The elution profile was programmed as follows: 0–5 min, 95:5 (A:B); 5–15 min, isocratic at 90:10; 15–30 min, linear gradient to 80:20; 30–45 min, isocratic at 80:20; followed by re-equilibration to initial conditions. The flow rate was set at 1.0 mL min^−1^, and the injection volume was 5 μL. All solvents were filtered through a 0.45 μm membrane filter and degassed before use. Tannic acid (Sigma-Aldrich, St. Louis, MO, USA) was used for calibration and quantification, and results were expressed in terms of tannic acid equivalents.

Total phenolic content was determined based on the modified Folin–Ciocalteu method, previously validated in our laboratory [29]. Briefly, 1 mL of ethanolic extract (equivalent to 1 mg of dry sample) or standard gallic acid solution was mixed with 1 mL of 10% Folin–Ciocalteu reagent, followed by the addition of 1 mL of 2% (*w*/*v*) sodium carbonate. The mixture was incubated in the dark for 90 min at room temperature. Absorbance was measured at 750 nm using a Spectramax i3 microplate reader (Molecular Devices, Sunnyvale, CA, USA). The concentration of phenolic compounds was calculated from a standard curve constructed with gallic acid, and the results were expressed as mg of gallic acid equivalents (GAE) per 100 g of dry sample (mg GAE 100 g^−1^).

### 2.9. Antioxidant Activities

For antioxidant analysis, finely powdered freeze-dried persimmon samples were extracted according to the protocol established by Baek et al. [29]. The antioxidant properties, including DPPH radical scavenging activity, Trolox-equivalent antioxidant capacity (ABTS), and ferric reducing antioxidant power (FRAP), were determined in triplicate. In addition, the reducing power assay was performed in triplicate following the method reported by Choi et al. [30].

### 2.10. Color Changes and Overall Sensory Evaluation

External fruit color parameters, including lightness (L*), redness (a*), yellowness (b*), and hue angle (h°), were measured on 15 fruits, with three measurements per fruit using a CR-400 Chroma Meter (Minolta, Tokyo, Japan). The total color difference (ΔE) before and after deastringency treatment was calculated as follows [31].

$$\Delta E=\sqrt{{\left(\Delta L^{*}\right)}^{2}+{\left(\Delta a^{*}\right)}^{2}+{\left(\Delta b^{*}\right)}^{2}}$$

where ΔL*, Δa* and Δb* represent the mean changes in each color parameter.

Overall sensory evaluation, including deastringency, flavor, color, sweetness, and texture, were assessed by a panel of 10 trained graduate students using a five-point scale ranging from 1 (poor) to 5 (excellent), as described by Choi et al. [32].

### 2.11. Statistical Analysis of Quality Parameters

Statistical analysis of quality parameters was conducted as follows. All data were expressed as means ± standard errors, and significant differences among treatments were determined by analysis of variance (ANOVA) using SAS statistical software (SAS/STAT^®^ 9.4; SAS Institute Inc., Cary, NC, USA) at a significance level of *p* < 0.05. When ANOVA indicated significance, Duncan’s multiple range test was applied for post hoc comparisons. For multivariate analysis, Pearson’s correlation analysis was performed to evaluate associations among the measured parameters. Prior to heatmap generation, VIP score calculation, and partial least squares discriminant analysis (PLS-DA), the dataset was normalized by median centering followed by autoscaling. Heatmaps visualization, VIP scores, and PLS-DA were performed using MetaboAnalyst 6.0 (https://www.metaboanalyst.ca/ (accessed on 11 August 2024)).

## 3. Results and Discussion

### 3.1. Assembly, Annotation, and DEGs

**Figure 1:**
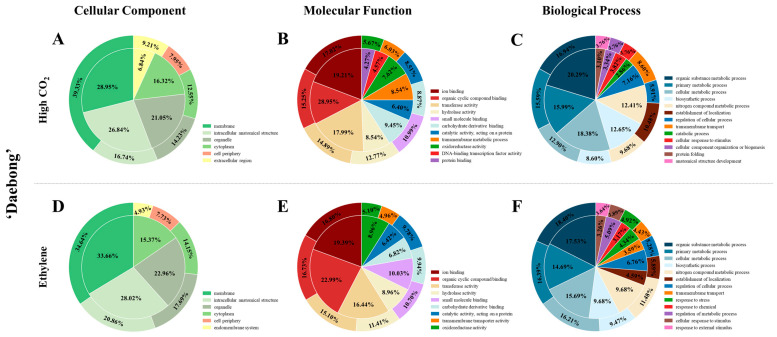
Summary of gene ontology (GO) analysis of differentially expressed genes (DEGs) in ‘Daebong’ persimmon under high CO_2_ (**A**–**C**) and ethylene (**D**–**F**) deastringency treatments. The analysis is categorized into cellular component (**A**,**D**), molecular function (**B**,**E**), and biological process (**C**,**F**). In the figure, the outer ring indicates upregulated genes, while the inner ring shows downregulated genes compared with the control. The results are based on comparisons made 1 day after high CO_2_ treatment and 4 days after ethylene treatment at 25 °C.

The summaries of raw, trimmed, and mapped data at harvest (control), and on the 1st and 4th days following deastringency treatments with high CO_2_ and ethylene at 25 °C are presented in Table 1. In this study, three ‘Daebong’ persimmon fruit transcriptome datasets were generated. The datasets were fruit at commercial harvest (control) and fruit at ‘ready to eat’ stage (high CO_2_-treated fruit on the 1st day and ethylene-treated fruit on the 4th day). The control, high CO_2_ and ethylene libraries produced an average of 67.20, 76.17 and 75.79 million reads, respectively, with corresponding mapped averages of 43.60, 44.37 and 47.46 million reads (Table 1) using a de novo approach. A total of 30,183 unigenes were identified with an average length of 1698 bp in the assembled sequences generated from RNA-Seq data. Table 1 also shows DEGs identified in comparisons of control fruits with those treated with high CO_2_ on the 1st day and treated with ethylene on the 4th day. Figure 1 summarizes the Gene Ontology (GO) categorization of differentially expressed genes (DEGs) identified in ‘Daebong’ persimmon after high CO_2_ and ethylene treatments. The GO terms were grouped into three major categories: cellular component, molecular function, and biological process. In the cellular component category, both up- and downregulated genes were predominantly related to the membrane and intracellular anatomical structures under both treatments, accounting for more than 50% of the annotated genes. This indicates that modifications in membrane-associated structures [21] and intracellular membrane decompartmentalization [33] are a key response during deastringency processes in persimmon. Cell membrane permeability changes can facilitate acetaldehyde accumulation in persimmon fruit tissues, thereby promoting tannin insolubilization during deastringency processes [11,34,35]. However, membrane permeability alterations are just one of several factors contributing to acetaldehyde accumulation. The main driver of this accumulation is anaerobic metabolism, which activates PDC and ADH pathways. These physiological changes are reflected in the differential expression of genes categorized by GO analysis. In the cellular component category, both up- and downregulated genes were mainly related to the membrane and intracellular anatomical structures under both treatments. For the molecular function category, a large proportion of DEGs were associated with ion binding and organic cyclic compound binding. Regarding the biological process category, organic substance metabolic process, primary metabolic process, and cellular metabolic process were the most enriched GO terms for both high CO_2_ and ethylene-treated fruits.

### 3.2. DEGs in the Comparison of High CO_2_ and Ethylene Treated vs. Control Persimmon Fruit

**Figure 2:**
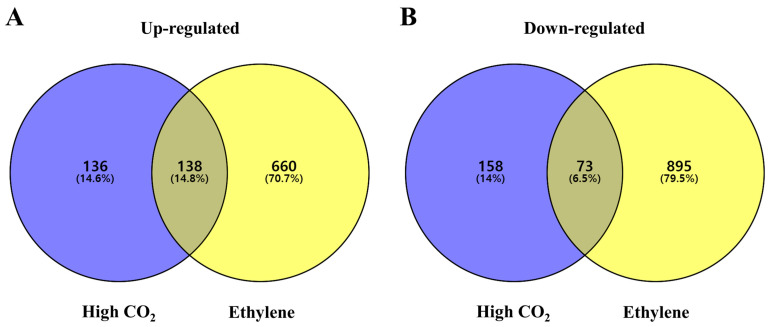
Numbers of commonly and exclusively expressed genes: (**A**) represents upregulated genes, and (**B**) represents downregulated genes, based on a log_2_ fold change greater than 2 and *p*-value < 0.05, during the comparison of high CO_2_ (at harvest vs. 1st day under high CO_2_), ethylene (at harvest vs. 4th day under ethylene in ‘Daebong’ astringent persimmon fruit.

DEGs were identified with a log_2_ fold change > 2 and *p* < 0.05 by comparing control fruit with high CO_2_- and ethylene-treated fruits. A total of 505 and 1766 unigenes were differentially expressed in high CO_2_- and ethylene-treated fruits, respectively (Table 1). Of these, 274 (29.4%) were upregulated and 231 (20.5%) were downregulated in high CO_2_-treated fruits, while 798 (85.5%) were upregulated and 968 (86%) were downregulated in ethylene-treated fruits (Table 1). Overall, ethylene treatment induced a larger number of DEGs compared to high CO_2_ treatment. As summarized in Table 1 and Figure 2, both high CO_2_ and ethylene treatments resulted in an overall increase in unigene expression compared to the control, with ethylene treatment inducing a larger number of DEGs. Venn diagram analysis revealed that in high CO_2_-treated fruits, 136 genes (14.6%) were exclusively upregulated and 158 genes (14%) were exclusively downregulated (Figure 2). In ethylene-treated fruits, 660 genes (70.7%) were exclusively upregulated and 895 genes (79.5%) were exclusively downregulated. In addition, 138 genes (14.8%) were commonly upregulated, and 73 genes (6.5%) were commonly downregulated under both treatments (Figure 2). The treatments induced higher unigene expression compared to the control, with ethylene treatment inducing more unigene expression than high CO_2_ treatment.

### 3.3. Soluble Tannin, Total Phenolics, and Genes Related to Deastringency

**Figure 3:**
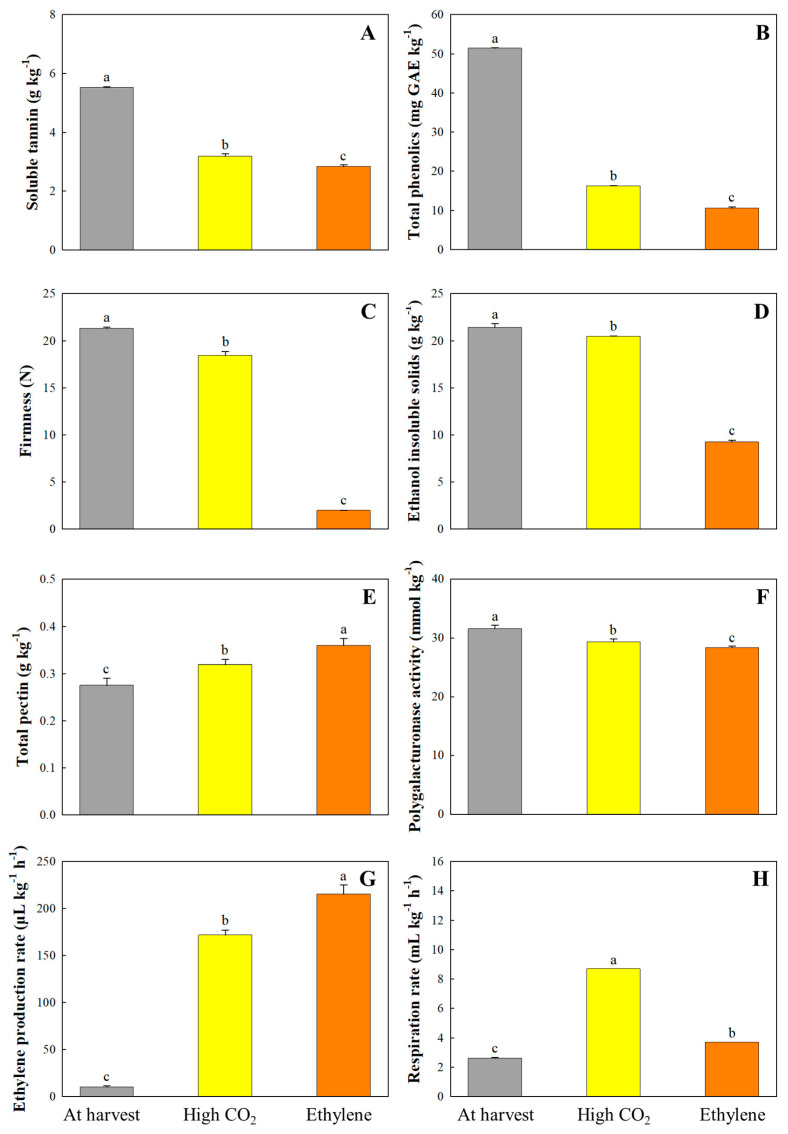
The soluble tannin (**A**), total phenolics (**B**), firmness (**C**), ethanol insoluble solids (EIS) (**D**), total pectin (**E**), polygalacturonase (PG) activity (**F**), ethylene production (**G**), and respiration rate (**H**) of the ‘Daebong’ astringent persimmon fruit at harvest, 1 day after high CO_2_ treatment and 4 days after ethylene treatment at 25 °C. Data are presented as mean ± standard errors (*n* = 15 for firmness; *n* = 3 for the other parameters). Different letters on the bars indicate significant differences between treatments at α = 0.05 with Duncan’s mean separation procedure.

Soluble tannins, a class of high molecular weight polyphenols with numerous hydroxyl groups, are primarily responsible for the astringent sensation in persimmon fruit [2]. In the present study, both high CO_2_ and ethylene treatments significantly decreased the soluble tannin content in ‘Daebong’ persimmons, thereby enhancing edibility (Figure 3). At harvest, the initial soluble tannin concentration was 5.52 g kg^−1^. This value declined to 3.18 g kg^−1^ after one day of high CO_2_ exposure and further decreased to 2.84 g kg^−1^ by the fourth day of ethylene treatment (Figure 3). The reduction in tannin content is attributed to acetaldehyde accumulation induced by anaerobic conditions during high CO_2_ treatment, which promotes the conversion of soluble tannins into insoluble forms, thus effectively mitigating astringency [2]. Meanwhile, Ethylene treatment promotes the transcription of ripening-related genes, including those associated with cell wall degradation and ethylene-responsive transcription factors (*ERFs*), which subsequently activate downstream genes such as pyruvate decarboxylase (*PDC*) and alcohol dehydrogenase (*ADH*). This regulatory cascade facilitates acetaldehyde accumulation and contributes to the polymerization and insolubilization of soluble tannins, thereby inducing a gradual loss of astringency during fruit ripening [21,36,37,38].

The total phenolic content in ‘Daebong’ persimmons was markedly reduced from 5.14 g kg^−1^ at harvest to 1.62 g kg^−1^ after one day of high CO_2_ exposure and continued to decrease to 1.06 g kg^−1^ by the fourth day following ethylene treatment. Although previous studies have reported a decrease in phenolic and tannin content during ripening or postharvest deastringency [39,40,41], recent comparative analyses suggest that these reductions may follow cultivar-specific regulatory strategies. For instance, high-phenolic cultivars such as ‘Rojo Brillante’ and ‘Rama Forte’ exhibit pronounced declines in tannin levels following CO_2_ or ethylene exposure, reflecting a rapid transition in their antioxidant and astringency profiles [37,41]. In contrast, other cultivars show more gradual or limited reductions, possibly linked to inherent differences in gene regulation of the phenylpropanoid pathway or polyphenol oxidase activity. Given the substantial decrease in both total phenolics and soluble tannins observed in ‘Daebong’ following deastringency treatment in this study, it is plausible that this cultivar shares a similar regulatory pattern with high-tannin, CO_2_, or ethylene-sensitive types. Such classification could provide useful insight into tailoring postharvest protocols depending on the biochemical and genetic predisposition of each cultivar.

A similar decline in tannin levels has been reported in ‘Rojo Brillante’ persimmons subjected to postharvest thermal treatment. For example, Cervera-Chiner et al. [31] demonstrated that drying at 40–45 °C reduced tannin content from approximately 30 g kg^−1^ DW at harvest to below 3 g kg^−1^ DW, effectively eliminating detectable astringency. This suggests that, regardless of treatment type be it gas exposure or temperature the critical reduction threshold for astringency lies around 3 g kg^−1^ DW.

To clarify the molecular mechanisms involved in persimmon deastringency, transcriptome profiling was conducted following treatments with high CO_2_ and ethylene. Both treatments resulted in a significant reduction in soluble tannin content, demonstrating effective deastringency (Figure 3). Additionally, distinct sets of DEGs were identified, with some genes common to both treatments and others specific to each. Among the commonly upregulated genes, sucrose synthase 2-like (*SuSy2*) and malate synthase, glyoxysomal (*MASY*) were notably induced (Table 2). Although both genes were upregulated under both CO_2_ and ethylene treatments, their functional relevance is particularly associated with enhanced glycolytic flux supporting anaerobic metabolism during CO_2_ treatment. Additionally, 1-aminocyclopropane-1-carboxylate oxidase (*ACO*) 1, a key enzyme in ethylene biosynthesis, and several ethylene-responsive transcription factors (e.g., *ERF12*, *ERF21*, *ERF115*, *ABR1*) were upregulated, indicating ethylene signal transduction involvement in both treatments (Table 2). Among the commonly upregulated genes, *UGT89B1-like* and *UGT75C1-like*, members of the UDP-glycosyltransferase family, may participate in modifying phenolic compounds through glycosylation. This process could decrease their solubility and contribute to the insolubilization of tannins during the deastringency process (Table 2). Furthermore, the common induction of *WRKY11/31*, NAC domain-containing proteins, and *MYB108* suggests their regulatory involvement in deastringency-related stress responses and phenolic metabolism (Table 2). These transcription factors may act as integrators of hormonal signaling (ethylene, jasmonate) and environmental stress, coordinating the transcriptional reprogramming required for astringency loss. In the CO_2_-specific transcriptome, several genes involved in anaerobic fermentation and redox metabolism, including *ADH 1/3*, peroxidase 5-like, and heat shock proteins, were exclusively upregulated. This indicates a metabolic shift towards hypoxic respiration and stress adaptation. Notably, *ERF14/26* and *WRKY24/46* were also induced specifically under CO_2_, further supporting their roles in coordinating hypoxia-induced deastringency (Table 3). In contrast, ethylene-specific upregulated genes included phenylalanine ammonia-lyase (*PAL*) and caffeic acid O-methyltransferase (*COMT*), which are involved in the phenylpropanoid pathway, potentially enhancing secondary metabolism during ethylene-induced ripening (Table 4). The up-regulation of *laccase 5* and *polygalacturonase-like* genes also implies restructuring of the cell wall and polymerization of phenolic compounds, possibly contributing to insolubilization of soluble tannins (Table 4). Interestingly, 4-coumarate—CoA ligase-like 1 (*4CL-like*) and *beta-amyrin 28-monooxygenase-like*, genes linked to lignin and triterpenoid biosynthesis, were commonly downregulated across both treatments, suggesting a suppression of lignification and secondary metabolic branches that may otherwise compete with tannin polymerization processes (Table 2). Overall, these results suggest that CO_2_ and ethylene treatments both activate distinct and overlapping regulatory networks for soluble tannin insolubilization and phenolic metabolism.

### 3.4. Firmness, EIS, Total Pectin, PG Activity, and Related Genes

At harvest, the firmness of ‘Daebong’ persimmon fruit was 21.31 N. At the ‘ready to eat’ stage, firmness was significantly retained in high CO_2_-treated fruit (18.44 N), whereas ethylene-treated fruit showed extensive softening, decreasing to 1.95 N (Figure 3). This pronounced reduction in firmness under ethylene treatment reflects the activation of ripening-associated processes, as previously reported in other climacteric fruits [42,43]. Changes in firmness were paralleled by variations in ethanol-insoluble solids (EIS), which dropped from 21.40 g kg^−1^ in the control to 9.23 g kg^−1^ in ethylene-treated fruit. The strong correlation between EIS and texture deterioration suggests that EIS can be a practical biochemical indicator for firmness during persimmon ripening [32]. In contrast, CO_2_-treated fruit retained higher EIS levels, aligning with the delayed softening. Total pectin content slightly increased during both treatments, reaching 0.32 g kg^−1^ (CO_2_) and 0.36 g kg^−1^ (ethylene), from 0.27 g kg^−1^ at harvest. Notably, this increase likely reflects pectin solubilization in the early stages of softening, as reported by Taira et al. [44]. However, polygalacturonase (PG) activity—a critical enzyme in pectin depolymerization—was reduced in both treatments, from 31.57 mmol kg^−1^ in the control to 29.34 mmol kg^−1^ (CO_2_) and 28.39 mmol kg^−1^ (ethylene), suggesting limited enzymatic degradation at the tested stages.

Transcriptomic analysis indicated that these physicochemical changes were closely linked to the expression of genes related to the cell wall. In CO_2_-treated fruit, notably, the up-regulation of *expansin-like A2* and xyloglucan endotransglucosylase/hydrolase (*XTH*) *23/33* indicates controlled cell wall remodeling without excessive disassembly, thereby contributing to tissue resilience and firmness maintenance. These genes are involved in reversible wall loosening, which enables flexibility while maintaining structural integrity (Table 3). In parallel, *peroxidase 5-like* and *peroxidase 51*, which are exclusively upregulated under CO_2_ treatment, may facilitate the oxidative cross-linking of phenolic compounds within the cell wall matrix (Table 3). This process is believed to enhance structural reinforcement by increasing cell wall rigidity, which improves resistance to softening. Such peroxidase-mediated stiffening mechanisms have been previously implicated in maintaining firmness in climacteric fruits during ripening [21]. On the other hand, the levels of transcripts for several cell wall-degrading enzymes were found to be lower. Pectate lyase 18, expansin A4 (*EXPA4*), and *beta-D-xylosidase* were significantly downregulated (Table 3), consistent with the observed suppression of PG activity. In particular, repression of *EXPA4*, which is typically upregulated during ripening to promote cell wall disassembly, likely plays a key role in restricting tissue softening in CO_2_-treated fruit. In contrast, ethylene treatment caused increases in transcripts of *polygalacturonase-like isoform X2*, *xyloglucan endotransglucosylase/hydrolase 10* and *11*, indicating activation of cell wall modification pathways during softening. However, the softening progression in ‘Daebong’ persimmons seems to require additional post-transcriptional or enzymatic regulation, as transcriptional upregulation alone may not completely account for the observed textural changes. These findings are consistent with previous observations in peach and persimmon, where CO_2_ treatment suppressed PG activity and preserved firmness through modulation of cell wall-related genes [6,45]. In contrast, ethylene treatment reduced firmness by regulating cell wall–modifying genes [17]. The observed retention of firmness in CO_2_-treated fruit may thus result from a combination of biochemical stabilization (e.g., EIS maintenance) and transcriptional regulation of cell wall-degrading enzymes. Ethylene-treated fruit, on the other hand, undergoes rapid softening, not necessarily through PG overexpression, but potentially due to the cumulative action of ripening-promoting regulators and ethylene-responsive transcription factors. Collectively, these results highlight the differential regulation of fruit texture by high CO_2_ and ethylene treatments, underscoring the potential to fine-tune postharvest quality through targeted modulation of associated genes. Both CO_2_ and ethylene treatments resulted in distinct transcriptional changes associated with cell wall remodeling, which were consistent with observed differences in fruit firmness, EIS, total pectin content, and PG activity. In the ethylene-treated fruits, firmness and EIS values markedly decreased, PG activity showed a slight reduction, and pectin content increased moderately. In contrast, high CO_2_-treated fruits maintained higher firmness and cellular resistance (Figure 3). At the transcriptional level, ethylene-specific induction of polygalacturonase-like genes (e.g., *polygalacturonase At1g48100-like*, *polygalacturonase*) was observed, aligning with the elevated PG activity and enhanced degradation of pectin components (Table 4). These genes are known to catalyze the depolymerization of homogalacturonan, contributing to middle lamella dissolution and tissue softening during fruit ripening and astringency removal. Additionally, the up-regulation of *laccase 5* and *laccase-15-like* may promote phenolic polymerization and cross-linking within the cell wall matrix, further contributing to loss of firmness through structural reorganization.

In contrast, several cell wall biosynthetic and remodeling genes were downregulated in both treatments, particularly in ethylene-treated fruits (Table 2). These include cellulose synthase-like proteins (*G3 isoforms*), *pectinesterase inhibitor 9-like*, and *beta-xylosidase-like* genes, which are essential for cell wall reinforcement and hemicellulose stability. Their suppression suggests attenuation of cell wall renewal and maintenance, potentially favoring wall loosening and cell separation. Notably, *expansin-like A2* and xyloglucan endotransglucosylase/hydrolase (*XTH*) genes were exclusively upregulated under CO_2_ treatment, indicating a unique remodeling response that may allow controlled loosening of the cell wall without extensive depolymerization (Table 3). Additionally, the CO_2_-specific up-regulation of *peroxidase 5-like* and *peroxidase 51* may reflect oxidative cross-linking of wall-bound phenolics, contributing to the retention of firmness through wall-strengthening mechanisms. From a hormonal regulation perspective, the up-regulation of ethylene-responsive transcription factors (*ERF14/21/115*, *WIN1-like*) and NAC domain-containing proteins (*NAC1*, *NAC2*) in both treatments supports their involvement in cell wall-related transcriptional reprogramming (Table 2). In particular, *WIN1-like* (homologous to Arabidopsis SHINE1), has been implicated in regulating cuticle and cell wall modification genes, potentially acting as a hub for ethylene-mediated wall loosening. Furthermore, the ethylene-specific induction of *MYB101*, *MYB108*, and *RAV1-like* transcription factors may contribute to the transcriptional regulation of wall-degrading enzymes, thereby facilitating cell wall disassembly and the softening process characteristic of ethylene-induced deastringency (Table 4). Under CO_2_ treatment, the down-regulation of genes such as Expansin A4 (*EXPA4*), GDSL esterase/lipase (*At1g54790-like*), and Xyloglucan O-acetyltransferase 4-like indicates a suppression of wall-loosening and pectin-modifying activities (Table 3). This transcriptional pattern likely contributes to the maintenance of cell wall rigidity and firmness during the deastringency process. Collectively, these findings indicate that while both CO_2_ and ethylene treatments activate cell wall modification pathways essential for deastringency, their regulatory outputs diverge: ethylene promotes active wall degradation and softening, whereas CO_2_ facilitates controlled remodeling coupled with oxidative wall stabilization, thereby preserving firmness.

### 3.5. Ethylene Production and Respiration Rates and Related Genes

At the ‘ready-to-eat’ stage, both ethylene production and respiration rates were markedly elevated in ‘Daebong’ persimmon compared to the harvest stage (Figure 3). Ethylene production increased from 9.97 μL kg^−1^ h^−1^ at harvest to 171.71 μL kg^−1^ h^−1^ after one day of high CO_2_ treatment and further rose to 215.42 μL kg^−1^ h^−1^ following four days of ethylene exposure. Likewise, the respiration rate increased from 2.61 mL kg^−1^ h^−1^ at harvest to 8.70 mL kg^−1^ h^−1^ in CO_2_-treated fruit and 3.70 mL kg^−1^ h^−1^ in ethylene-treated fruit, respectively. These metabolic responses are likely triggered by external stress signals induced by the deastringency treatments, particularly high CO_2_, which is known to cause a temporary stimulation of respiratory activity [46]. Despite this initial metabolic activation, high CO_2_-treated fruits retained firmness, which may be attributed to the repression of cell wall-degrading enzyme activity [45]. Although both metabolic indicators were transiently elevated by CO_2_ treatment, fruit firmness was largely preserved, especially in comparison to ethylene-treated samples. This paradoxical outcome suggests the presence of transcriptional and metabolic controls that uncouple increased metabolic activity from downstream softening responses. For example, the AP2-like ethylene-responsive transcription factor (*At1g16060*) was specifically downregulated in CO_2_-treated fruit (Table 3). As a key regulator of ethylene signaling, this gene controls the expression of cell wall-modifying enzymes, such as expansins and pectinases. Its repression implies a blockade of ethylene-mediated transcriptional cascades, thereby attenuating softening processes even in the presence of ethylene biosynthetic activity. Moreover, the up-regulation of mitochondrial aspartate aminotransferase (*AAT*) may reflect a metabolic shift toward amino acid interconversion, functioning as a buffer against excess respiratory flux and redox imbalance. In contrast, the down-regulation of pyrophosphate-energized vacuolar membrane proton pump suggests a possible reduction in vacuolar acidification and energy-dependent solute transport, which could restrain enzymatic activities involved in cell wall disassembly. Collectively, these molecular adjustments underscore that the maintenance of firmness under CO_2_ treatment is not merely due to suppressed ethylene biosynthesis, but rather due to targeted inhibition of ethylene signaling pathways and selective repression of respiration-linked softening mechanisms.

Genes involved in ethylene biosynthesis and signaling were exclusively upregulated under ethylene treatment. Notably, 1-aminocyclopropane-1-carboxylate synthase (*ACS*), a key rate-limiting enzyme in ethylene biosynthesis, was strongly induced, providing a direct molecular explanation for the observed ethylene surge (Table 4). In addition, the up-regulation of EIN3-binding F-box protein 1-like and ethylene-overproduction protein 1—key components of the ethylene signal transduction cascade—suggests enhanced downstream transcriptional activation of ethylene-responsive genes. Multiple AP2/ERF family transcription factors, including *ERF061*, *ERF113-like*, *ERF008-like*, *RAP2-1-like*, and *RAP2-7-like*, were also upregulated, indicating a robust activation of ethylene-mediated transcriptional networks that likely regulate cell wall-degrading enzymes such as polygalacturonases and pectinases (Table 4). This transcriptional reprogramming is aligned with the accelerated softening observed in ethylene-treated fruit. Furthermore, genes associated with respiration and energy metabolism were specifically induced. These include pyruvate decarboxylase 1-like, ATP-dependent 6-phosphofructokinase 4, mitochondrial phosphate carrier protein 3, and respiratory burst oxidase homolog A-like, which are integral to glycolysis, mitochondrial ATP synthesis, and redox regulation (Table 4). Their induction supports the notion that ethylene treatment stimulates energy-demanding processes during ripening.

### 3.6. Antioxidant Activities and Stress-Related Genes

**Figure 4:**
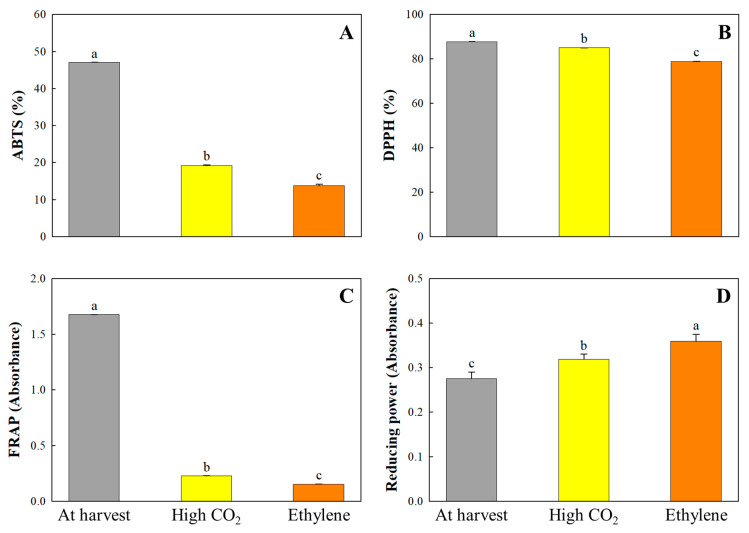
Trolox-equivalent antioxidant capacity (ABTS) (**A**), DPPH (2,2-di-phenyl-1-picrylhydrazyl) radical scavenging capacity (**B**), ferric-reducing antioxidant power (FRAP) (**C**), and reducing power (**D**) of the ‘Daebong’ astringent persimmon fruit at harvest, 1 day after high CO_2_ treatment and 4 days after ethylene treatment at 25 °C. Data are presented as mean ± standard errors (*n* = 3). Different letters on the bars indicate significant differences between treatments at α = 0.05 with Duncan’s mean separation procedure.

Significant reductions in antioxidant activity were observed in ‘Daebong’ persimmons after both high CO_2_ and ethylene deastringency treatments, evaluated using ABTS, DPPH, FRAP, and reducing power assays. Notably, ABTS scavenging activity declined from 47.07% at harvest to 19.18% after CO_2_ treatment and further to 13.75% under ethylene treatment (Figure 4), indicating that ethylene induces greater oxidative deterioration. Despite overall declines, CO_2_-treated fruits consistently retained higher antioxidant activities than ethylene-treated counterparts, suggesting treatment-specific modulation of stress and redox responses. These trends paralleled changes in soluble tannins and total phenolic content, reinforcing their role as key contributors to the antioxidant profile, as previously reported by Katsube et al. [47] and Denev and Yordanov [40].

Transcriptomic results showed that high CO_2_ treatment significantly upregulated several genes related to oxidative stress. For example, glutathione S-transferase U17-like (*GSTU17*) and other GST isoforms were clearly upregulated under CO_2_ (Table 3), indicating increased detoxification of reactive oxygen species (ROS). The upregulation of peroxidase 5-like, peroxidase 51, and alcohol dehydrogenase 1 and 3 further indicates activation of enzymatic scavenging systems that reduce lipid peroxidation and cellular damage (Table 3). Heat shock proteins (HSPs) such as HSP70, 22 kDa HSP, and several small HSPs were exclusively induced, demonstrating that CO_2_ triggered a broad cytoprotective response to maintain protein stability under hypoxic stress (Table 3). Moreover, the activation of WRKY and NAC transcription factors—key regulators of abiotic stress and redox homeostasis—suggests a coordinated transcriptional response to maintain cellular stability. The presence of universal stress protein A (USP-A) and jasmonate-induced oxygenase (*JOX2*) indicates hormonal interactions between jasmonate and ethylene, thereby enhancing defense responses. These molecular adaptations suggest that antioxidant activity remains partially preserved during CO_2_ treatment, despite the metabolic stress. In contrast, ethylene treatment failed to elicit a comparable stress-mitigating response. While several ethylene-responsive transcription factors were induced, the genes directly related to ROS detoxification and antioxidant defense were notably underrepresented or even suppressed For example, the down-regulation of lipoxygenase (*LOX2-1*) and peroxidase 12-like (*POD5*) in response under ethylene (Table 4) indicates reduced ROS signaling and control of lipid peroxidation, which may lead to the observed decrease in antioxidant activity. Furthermore, the limited expression of heat shock proteins (HSPs) and glutathione S-transferases (GSTs) in ethylene-treated samples underscores the treatment’s reduced ability to maintain cellular redox balance during the ripening process. These findings suggest that high CO_2_ treatment induces a more comprehensive and effective oxidative stress response than ethylene, resulting in improved preservation of antioxidant function and metabolic resilience during deastringency. The transcriptomic results indicate that hypoxia-induced stress under CO_2_ enhances protective gene expression, offering the dual benefits of astringency removal and retention of nutritional quality.

### 3.7. Color Changes, Overall Sensory Quality, and the Related Genes

**Figure 5:**
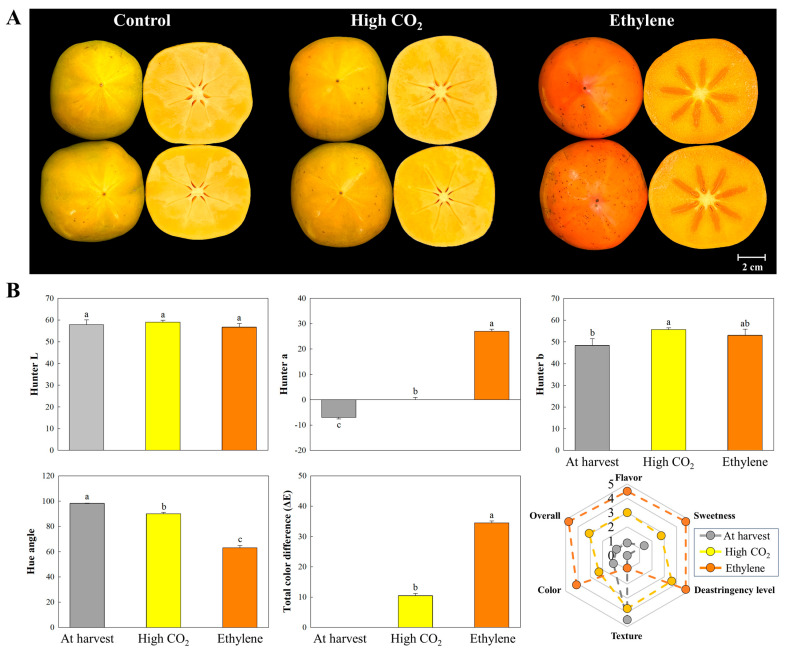
Representative photos (**A**), color parameters (hunter L, a, b values, hue angle, total color difference), and sensory evaluation (**B**) of ‘Daebong’ astringent persimmon fruit at harvest, 1 day after deastringency treatment with high CO_2_, and 3 days after deastringency treatment with ethylene at 25 °C. Data are presented as means ± standard errors (*n* = 15 for color values; *n* = 10 for sensory evaluation). Different letters on the bars indicate significant differences between treatments at α = 0.05 with Duncan’s mean separation procedure.

At the ready-to-eat stage, fruits treated with ethylene showed a significantly greater total color difference (ΔE = 34.45) compared to those treated with CO_2_ (ΔE = 10.44), indicating more advanced ripening and external pigmentation (Figure 5). In ethylene-treated fruit, the overall sensory quality score significantly increased from 0.9 at harvest to 4.75 by day 4, while CO_2_-treated fruit reached a moderate score of 3.1 on day 1. These findings indicate that ethylene not only improves visual qualities but also increases palatability, likely by reducing soluble tannins and significantly softening, as previously reported by Choi et al. [32]. In contrast, CO_2_ treatment moderately enhanced color and flavor while preserving firmness, underscoring its role in extending postharvest shelf life.

Transcriptomic profiling revealed specific regulatory mechanisms related to the treatment that underlies these phenotypic outcomes. In fruit treated with ethylene, genes related to pigment biosynthesis and the formation of volatile compounds were significantly upregulated. Specifically, 9-cis-epoxycarotenoid dioxygenase (*NCED2*), which catalyzes the oxidative cleavage of carotenoids and contributes to both ABA biosynthesis and color change, was strongly induced. Likewise, Phenylalanine ammonia-lyase (*PAL*), a key enzyme in the phenylpropanoid pathway, was upregulated, which supports the production of flavonoid precursors that affect both color and taste. Additionally, *alcohol acyltransferase 9* and *monooxygenase 2-like*, which are involved in the biosynthesis of esters and aroma compounds, were upregulated. This indicates an enhancement of fruit flavor and volatile profiles. The activation of these metabolic pathways corresponds with the high sensory acceptance observed in ethylene-treated fruit.

In contrast, the fruit treated with CO_2_ showed a more controlled transcriptional response, indicating a strategic adjustment of secondary metabolism and structural maintenance instead of accelerating ripening (Table 3). Notably, protein *TIFY 9-like* and *jasmonate-induced oxygenase 1-like isoform X1*, exclusively upregulated under CO_2_ treatment, suggest involvement of the jasmonate signaling pathway, which is known to modulate stress tolerance and may indirectly influence flavor and firmness retention during postharvest treatment. Moreover, the induction of *expansin-like A2* and *wall-associated receptor kinase-like 10 isoform X5* suggests a regulated cell wall remodeling process, which may facilitate essential but slight changes needed for moderate color development without affecting fruit integrity. Furthermore, *universal stress protein A-like protein* and *glutathione S-transferase U17-like isoform X1* were upregulated, suggesting activation of redox balancing and antioxidant defense systems that could preserve pigment stability and prevent excessive tissue softening under hypoxic CO_2_ conditions. These expression patterns indicate that high CO_2_ treatment promotes a unique transcriptional adjustment aimed at maintaining texture and extending shelf life while allowing limited quality enhancement.

Overall, these transcriptional dynamics suggest that ethylene enhances consumer-preferred attributes by activating biosynthetic genes related to color and flavor, while high CO_2_ preserves structural integrity and influences flavor through the stabilization of secondary metabolites. These differing strategies provide complementary methods for optimizing persimmon postharvest quality based on market needs.

### 3.8. Partial Least Squares-Discriminant and Correlation Analysis

**Figure 6:**
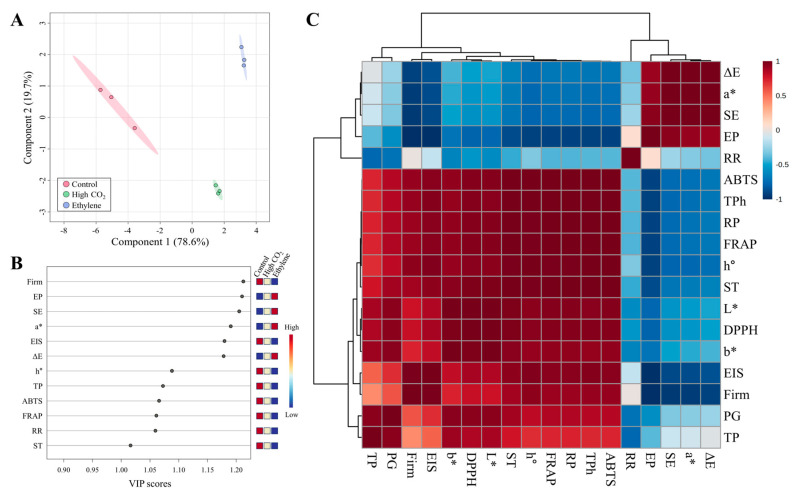
Partial least squares-discriminant analysis (PLS-DA) scores plot (**A**); variable importance in projection (VIP) scores plot (**B**); correlation heatmap (**C**) of the observed parameters in ‘Daebong’ astringent persimmon fruit at harvest, 1 day after deastringency treatment with high CO_2_, and 3 days after deastringency treatment with ethylene at 25 °C. ΔE, a*, SE, EP, RR, ABTS, TPh, RP, FRAP, h°, ST, L*, DPPH, b*, EIS, RR, Firm, PG, and, TP, represent color difference, Hunter a value, sensory evaluation, ethylene production rate, respiration rate, 2,2′-azino-bis(3-ethylbenzothiazoline-6-sulfonic acid), total phenols, reducing power, ferric reducing antioxidant power, hue angle, soluble tannin, Hunter L value, α-diphenyl-β-picrylhydrazyl, Hunter b value, ethanol insoluble solids, firmness, polygalacturonate activity, and total pectin, respectively. The heatmap’s red and blue boxes show positive and negative correlations.

The Partial least squares-discriminant (PLS-DA) score plot (Figure 6A) clearly distinguished the control, high CO_2_, and ethylene treatment groups along Component 1 (78.6% of the total variance) and Component 2 (10.7%). This separation reflects pronounced differences in quality attributes among the treatments, with both high CO_2_ and ethylene groups distinctly segregated from the control. Such clustering strongly suggests that the postharvest treatments induced specific metabolic and color changes associated with degreening processes.

VIP score analysis (Figure 6B) revealed that the most influential variables for treatment discrimination, in descending order, were firmness, ethylene production rate (EP), sensory evaluation (SE), a*, ethanol insoluble solids (EIS), ΔE, h°, and total phenolics (TP). These parameters are closely related to tissue integrity, pigment changes, and the accumulation of antioxidant compounds, serving as critical indicators for assessing quality retention during deastringency. Firmness and EIS, in particular, were strongly associated with quality maintenance in the CO_2_-treated group, supporting previous findings that CO_2_ treatment preserves firmness by suppressing cell wall degradation [45].

Pearson’s correlation analysis (Figure 6C) demonstrated a remarkably strong positive correlation between firmness and EIS (r = 0.990), highlighting a close link between cellular structural integrity and physical firmness. Firmness also showed strong positive correlations with color stability (h°, r = 0.939) and TP (r = 0.920), while exhibiting strong negative correlations with maturity-associated parameters such as EP (r = −0.982) and a* (r = −0.935). These patterns indicate that firmness loss is directly associated with accelerated color shifts and surface property changes, indicative of ripening.

Antioxidant activity was found to be tightly linked with total phenolics. TP was almost perfectly correlated with FRAP (r = 0.9997) and ABTS (r = 0.9998), while soluble tannin content also showed strong correlations with FRAP (r = 0.9966) and ABTS (r = 0.9968). These findings suggest that antioxidant capacity is primarily determined by the phenolic fraction, particularly soluble tannins. Regarding colorimetric indices, a* was highly correlated with total color change (ΔE, r = 0.998) and surface color uniformity (SE, r = 0.994), while b* was strongly correlated with lightness (L*, r = 0.994). Collectively, these results provide mechanistic insights into the maintenance of color stability in high CO_2_-treated fruit and the rapid pigment alterations observed in the ethylene-treated group. Moreover, the correlation patterns identified in Figure 6A–C offer a quantitative basis for developing non-destructive predictive models and designing targeted quality management strategies during storage and distribution.

## 4. Conclusions

This study showed that both high CO_2_ and ethylene treatments effectively reduced the soluble tannin content in ‘Daebong’ persimmons, aiding in deastringency. However, the effects on fruit quality traits, especially firmness, varied significantly. Fruits treated with high levels of CO_2_ maintained their firmness despite increased ethylene production and respiration rates. This suggests a role for transcriptional repression of ethylene signaling (*AP2-like ERFs*), reduced vacuolar transport activity, and enhanced antioxidant defense mechanisms. The activation of hypoxia-related genes and ROS-scavenging enzymes under CO_2_ treatment indicates an adaptive strategy for stress mitigation that maintains structural and biochemical stability. In contrast, treatment with ethylene initiated a broader transcriptional reprogramming, including the up-regulation of *ACS*, *polygalacturonase*, *ERF* genes, and genes related to energy metabolism. Collectively, these changes promoted rapid softening, development of pigments, and enhancement of sensory qualities. These varying responses underscore the intricate relationship between hormonal signaling, cell wall metabolism, and oxidative stress pathways during the deastringency of persimmons. The transcriptomic and biochemical responses specific to the cultivar described in this study offer a detailed molecular framework for enhancing postharvest treatment protocols for astringent persimmons. Using high CO_2_ treatment to remove astringency while preserving firmness and antioxidant capacity offers an effective strategy for maintaining postharvest quality in PCA-type cultivars like ‘Daebong’.

## Figures and Tables

**Table 1:** Summary of sequencing data, including raw, trimmed, and mapped reads for ‘Daebong’ persimmon fruits at harvest (control) and after deastringency treatments with high CO_2_ (1st day) and ethylene (4th day) at 25 °C. It also presents the number of differentially expressed genes (DEGs) identified in comparisons between the control and treated fruits.

Treatment	Raw Data	Trimmed Data	Mapped Reads	Mapping Rate (%)	Number of DEGs (Control vs. Treatments) *p* < 0.05, $\left|{log}_{2}\;Fold\;Change\right|\;\geq \;2$		
Control (at harvest)	67,200,370	64,955,644	43,602,204	67.1	Up	Down	Total
High CO_2_ (1 day)	76,167,104	72,745,056	44,372,198	61	274	231	505
Ethylene (4 day)	75,785,722	73,072,096	47,458,880	64.9	798	968	1766

**Table 2:** List of DEGs in ‘Daebong’ astringent persimmon cultivar (commonly) in the comparison of high CO_2_ and ethylene treated vs. control.

Gene ID	Gene Descriptions	Log_2_ Fold Change		Gene Functions
		High CO_2_	Ethylene	
Upregulated				
LOC127802412	*F-box protein PP2-B15-like*	12.26	13.86	Protein binding
AID51426.1	*ethylene response factor 21*	11.63	8.04	
LOC127797932	*flavanone 3-dioxygenase 3*	10.30	6.55	
LOC127803199	*PI-PLC X domain-containing protein At5g67130-like*	9.85	13.57	Posphoric diester hydrolase activity
LOC127810275	*F-box protein VBF-like*	9.15	7.60	Potein binding
LOC127794125	*1-aminocyclopropane-1-carboxylate oxidase 1*	9.11	11.64	
LOC127790996	*ethylene-responsive transcription factor ERF115-like*	9.05	9.49	DNA-binding transcription factor activity
LOC127798805	*ethylene-responsive transcription factor ABR1-like isoform X2*	8.16	7.20	DNA-binding transcription factor activity
LOC127802859	*lysine histidine transporter-like 8*	8.14	6.93	
LOC127812266	*sucrose synthase 2-like isoform X2*	8.13	5.18	Sucrose synthase activity
LOC127802270	*receptor-like protein kinase 7*	7.91	6.35	Potein kinase activity
PSR89067.1	*Membrane-associated kinase regulator*	7.91	8.97	
LOC127803148	*calcium-binding protein CML46*	7.63	4.57	Calcium ion binding
LOC127801080	*E3 ubiquitin-protein ligase ATL31-like*	7.54	8.88	
LOC127798535	*E3 ubiquitin-protein ligase RGLG5-like*	7.44	9.60	
LOC127795244	*malate synthase*, *glyoxysomal*	7.17	13.89	Malate synthase activity
LOC127808940	*mitogen-activated protein kinase kinase kinase 5-like isoform X1*	7.12	5.93	ATP binding
LOC127797657	*NAC domain-containing protein 1*	6.72	6.49	DNA binding
AID51417.1	*ethylene response factor 12*	6.33	5.72	
LOC127790200	*cationic peroxidase 1-like*	6.17	7.09	Peroxidase activity
LOC127787838	*galacturonosyltransferase-like 9*	6.09	6.17	Glycosyltransferase activity
LOC127809143	*cytochrome P450 86B1-like*	5.70	11.06	Oxidoreductase activity, acting on paired donors, with incorporation or reduction of molecular oxygen
PSR99923.1	*3-isopropylmalate dehydratase large subunit like*	5.67	10.58	
AEC11088.1	*MYB transcription factor PA1*	5.54	10.13	
LOC127797399	*NDR1/HIN1-like protein 13*	5.23	5.39	
LOC127809456	*transcription factor MYB108-like*	5.17	6.15	DNA-binding transcription factor activity
LOC127808889	*WRKY transcription factor 31*	5.06	8.36	DNA-binding transcription factor activity
LOC127801120	*L-ascorbate oxidase homolog*	4.98	4.53	Oxidoreductase activity
LOC127789678	*plant cysteine oxidase 1-like*	4.82	6.68	Oxidoreductase activity, acting on single donors with incorporation of molecular oxygen, incorporation of two atoms of oxygen
LOC127790398	*cytochrome P450 76A1-like*	4.40	11.89	Oxidoreductase activity, acting on paired donors, with incorporation or reduction of molecular oxygen
QGV56724.1	*WRKY transcription factor 15*	4.33	3.53	
LOC127801139	*beta-glucosidase 11-like isoform X1*	4.31	6.55	Beta-glucosidase activity
AZL19548.1	*transcription factor WRKY11*	4.30	5.83	
LOC127792859	*flavonol 3-O-glucosyltransferase UGT89B1-like*	4.30	4.68	UDP-glycosyltransferase activity
LOC127811263	*UDP-glycosyltransferase 75C1-like*	4.11	6.04	UDP-glycosyltransferase activity
LOC127795520	*ethylene-responsive transcription factor WIN1-like*	3.51	8.57	DNA-binding transcription factor activity
LOC127800536	*NAC domain-containing protein 2*	3.24	3.20	DNA binding
**Downregulated**				
LOC127787223	*pectinesterase inhibitor 9-like*	−7.66	−5.35	Enzyme inhibitor activity
LOC127812875	*cellulose synthase-like protein G3 isoform X4*	−7.45	−5.02	Cellulose synthase (UDP-forming) activity
LOC127800039	*germin-like protein subfamily 1 member 11*	−7.14	−6.30	Manganese ion binding
LOC127806638	*wax ester synthase/diacylglycerol acyltransferase 5-like*	−6.67	−12.37	Long-chain-alcohol O-fatty-acyltransferase activity
LOC114274781	*E3 ubiquitin-protein ligase MARCH10*	−6.15	−7.31	Zinc ion binding
LOC127810041	*beta-xylosidase/alpha-L-arabinofuranosidase 2-like isoform X2*	−6.00	−8.57	Alpha-L-arabinofuranosidase activity
LOC127812875	*cellulose synthase-like protein G3 isoform X2*	−5.94	−7.63	Cellulose synthase (UDP-forming) activity
LOC127808982	*berberine bridge enzyme-like 15*	−5.75	−7.91	Oxidoreductase activity
LOC127807607	*thaumatin-like protein 1*	−5.51	−8.67	
LOC127812875	*cellulose synthase-like protein G3 isoform X1*	−5.30	−6.95	Cellulose synthase (UDP-forming) activity
LOC127796807	*4-coumarate--CoA ligase-like 1*	−5.12	−4.94	Long-chain fatty acid-CoA ligase activity
PSR95878.1	*Pre-neck appendage protein*	−5.08	−4.36	
LOC127809129	*boron transporter 2*	−4.51	−5.50	Solute:inorganic anion antiporter activity
KAI7982608.1	*putative pectinesterase/pectinesterase inhibitor 40*	−4.20	−6.32	
LOC109009267	*expansin-A15 isoform X1*	−4.07	−8.39	
LOC127796063	*beta-amyrin 28-monooxygenase-like*	−3.96	−6.55	Oxidoreductase activity, acting on paired donors, with incorporation or reduction of molecular oxygen
LOC127803927	*cytochrome P450 71AU50-like isoform X3*	−3.77	−6.86	Oxidoreductase activity, acting on paired donors, with incorporation or reduction of molecular oxygen
PSS00292.1	*Developmental and secondary metabolism regulator veA like*	−3.73	−4.15	
LOC127800637	*serine/threonine-protein kinase WNK9 isoform X1*	−3.72	−6.83	Protein serine/threonine kinase activity
LOC127805784	*protein STRICTOSIDINE SYNTHASE-LIKE 4-like isoform X2*	−3.64	−6.73	Strictosidine synthase activity
LOC127813526	*glucomannan 4-beta-mannosyltransferase 2*	−3.61	−8.01	Mannan synthase activity
LOC127806942	*NADPH-dependent oxidoreductase 2-alkenal reductase-like*	−3.59	−4.54	Oxidoreductase activity, acting on the CH-CH group of donors, NAD or NADP as acceptor
LOC127798462	*IAA-amino acid hydrolase ILR1-like 1*	−3.37	−9.56	IAA-Ala conjugate hydrolase activity

**Table 3:** List of DEGs in ‘Daebong’ astringent persimmon cultivar (exclusively) in the comparison of high CO_2_ treated vs. control.

Gene ID	Gene Descriptions	Log_2_ Fold Change	Gene Functions
Upregulated			
LOC127810272	*peroxidase 5-like*	11.28	Peroxidase activity
LOC127799450	*22.0 kDa class IV heat shock protein-like*	9.89	Unfolded protein binding
LOC127803768	*17.5 kDa class I heat shock protein-like*	9.74	Unfolded protein binding
LOC127808179	*ethylene-responsive transcription factor ERF014-like*	9.22	DNA-binding transcription factor activity
LOC127797208	*glutathione S-transferase U17-like isoform X1*	9.11	Glutathione transferase activity
LOC127796264	*protein TIFY 9-like*	7.34	
LOC127804909	*xyloglucan endotransglucosylase/hydrolase protein 23*	6.80	Xyloglucan:xyloglucosyl transferase activity
LOC127800824	*aspartic proteinase GIP2*	6.78	Aspartic-type endopeptidase activity
PSS29393.1	*Mitochondrial fission regulator like*	6.77	
LOC127797673	*xyloglucan endotransglucosylase/hydrolase protein 33*	6.72	Xyloglucan:xyloglucosyl transferase activity
LOC127795131	*17.3 kDa class II heat shock protein-like*	6.72	Unfolded protein binding
LOC127808826	*alcohol dehydrogenase 1*	6.42	S-(hydroxymethyl)glutathione dehydrogenase [NAD(P)+] activity
LOC127790650	*(−)-isopiperitenol/(−)-carveol dehydrogenase*, *mitochondrial-like*	6.31	
LOC127796562	*class I heat shock protein*	6.29	Unfolded protein binding
LOC127806544	*heat shock 70 kDa protein*	6.26	Heat shock protein binding
LOC127810698	*2-oxoglutarate-dependent dioxygenase 19-like*	6.12	
LOC127793595	*pectinesterase/pectinesterase inhibitor 41*	6.11	Pectinesterase inhibitor activity
LOC127788897	*aspartate aminotransferase*, *mitochondrial-like isoform X2*	5.75	Pyridoxal phosphate binding
LOC127791465	*cytochrome P450 72A397-like*	5.68	Oxidoreductase activity, acting on paired donors, with incorporation or reduction of molecular oxygen
LOC127791460	*glucose-6-phosphate/phosphate translocator 2*, *chloroplastic-like*	5.66	Triose-phosphate transmembrane transporter activity
KAI8032162.1	*Peroxidase 51*	5.62	
LOC127792124	*1-aminocyclopropane-1-carboxylate oxidase*	5.44	
LOC127797235	*F-box protein SKIP28*	5.09	Protein binding
LOC127787406	*wall-associated receptor kinase-like 20*	4.99	Protein serine/threonine kinase activity
LOC127790698	*cytosolic sulfotransferase 12-like*	4.90	Sulfotransferase activity
LOC127806329	*cucumber peeling cupredoxin-like*	4.87	Electron transfer activity
LOC127800236	*universal stress protein A-like protein*	4.83	
LOC127799949	*alcohol dehydrogenase 3*	4.71	S-(hydroxymethyl)glutathione dehydrogenase [NAD(P)+] activity
LOC127805737	*jasmonate-induced oxygenase 1-like isoform X1*	4.66	
ASL69240.1	*ethylene response factor 26*	4.55	
LOC127793838	*protein TIFY 10A-like*	4.45	
LOC127794477	*wall-associated receptor kinase-like 10 isoform X5*	4.39	Polysaccharide binding
LOC127789995	*NADPH-dependent aldo-keto reductase*, *chloroplastic-like*	4.22	Aldose reductase (NADPH) activity
LOC127801110	*WRKY transcription factor 46*	4.11	DNA-binding transcription factor activity
LOC127801209	*expansin-like A2*	4.09	
LOC127799270	*glutathione S-transferase*	4.06	Glutathione transferase activity
LOC127793268	*endoplasmic reticulum oxidoreductin-1-like*	4.00	Thiol oxidase activity
LOC127792628	*epidermis-specific secreted glycoprotein EP1-like*	3.66	
LOC127789222	*putative receptor protein kinase ZmPK1*	3.66	Protein serine/threonine kinase activity
LOC127813273	*WRKY transcription factor WRKY24*	3.63	DNA-binding transcription factor activity
Q8S932.1	*1-aminocyclopropane-1-carboxylate oxidase*	3.62	
LOC127808277	*putative 12-oxophytodienoate reductase 11 isoform X1*	3.18	Oxidoreductase activity
**Downregulated**			
LOC127795923	*pectate lyase 18*	−8.67	Pectate lyase activity
LOC127799002	*putative beta-D-xylosidase*	−8.10	Xylan 1,4-beta-xylosidase activity
KAF5729599.1	*Expansin A4 ALPHA 1.6 EXPA4*	−8.08	
LOC127792836	*nucleobase-ascorbate transporter 4*	−7.20	Transmembrane transporter activity
LOC127787055	*monothiol glutaredoxin-S9-like*	−7.01	
LOC127792213	*cytochrome P450 724B1-like*	−6.77	Oxidoreductase activity, acting on paired donors, with incorporation or reduction of molecular oxygen
LOC127795951	*GDSL esterase/lipase 1-like isoform X5*	−5.46	Hydrolase activity, acting on ester bonds
LOC127793870	*AP2-like ethylene-responsive transcription factor At1g16060*	−5.33	DNA-binding transcription factor activity
LOC127800698	*aspartyl protease AED3*	−4.97	Aspartic-type endopeptidase activity
LOC127803129	*serine carboxypeptidase-like 25*	−4.90	Serine-type carboxypeptidase activity
PSS05082.1	*Phenylalanine ammonia-lyase*	−4.82	
LOC127812252	*stemmadenine O-acetyltransferase-like*	−4.73	
LOC127795350	*aspartyl protease AED3-like*	−4.61	Aspartic-type endopeptidase activity
LOC127813223	*nudix hydrolase 8-like*	−4.60	NADH pyrophosphatase activity
LOC127798278	*peroxidase P7-like*	−4.51	Peroxidase activity
LOC127790066	*pyrophosphate-energized vacuolar membrane proton pump-like*	−4.40	Diphosphate hydrolysis-driven proton transmembrane transporter activity
LOC127786991	*GDSL esterase/lipase At1g54790-like*	−4.33	Hydrolase activity, acting on ester bonds
LOC127793738	*beta-amyrin 28-monooxygenase-like*	−4.18	Oxidoreductase activity, acting on paired donors, with incorporation or reduction of molecular oxygen
LOC127795833	*cytochrome P450 736A117-like isoform X1*	−3.96	Oxidoreductase activity, acting on paired donors, with incorporation or reduction of molecular oxygen
LOC127809955	*glycosyl hydrolase 5 family protein-like*	−3.94	Hydrolase activity, hydrolyzing O-glycosyl compounds
AXQ60485.1	*acetaldehyde dehydrogenase*	−3.75	
LOC127787809	*UPF0481 protein At3g47200-like isoform X1*	−3.60	
LOC127793571	*7-deoxyloganetic acid glucosyltransferase-like*	−3.48	UDP-glycosyltransferase activity
LOC127791330	*NAC domain-containing protein 72-like*	−3.44	DNA binding
GFZ19603.1	*glycosyl hydrolase family protein*	−3.26	
LOC127799077	*xyloglucan O-acetyltransferase 4-like*	−3.23	O-acetyltransferase activity

**Table 4:** List of DEGs in ‘Daebong’ astringent persimmon cultivar (exclusively) in the comparison of ethylene treated vs. control.

Gene ID	Gene Descriptions	Log_2_ Fold Change	Gene Functions
Upregulated			
GFS33599.1	*laccase 5*	14.88	
LOC127812189	*stemmadenine O-acetyltransferase-like*	14.03	
KAH9668398.1	*Endonuclease*	12.98	
LOC127792808	*9-cis-epoxycarotenoid dioxygenase NCED2*, *chloroplastic*	12.97	Carotenoid dioxygenase activity
OVA11305.1	*Cyclin PHO80-like*	12.67	
LOC127812613	*metalloendoproteinase 4-MMP-like*	11.50	Metalloendopeptidase activity
LOC127803872	*indole-3-pyruvate monooxygenase YUCCA7*	11.09	N,N-dimethylaniline monooxygenase activity
LOC127792678	*glutathione S-transferase parC isoform X2*	11.05	Glutathione transferase activity
LOC127795217	*EIN3-binding F-box protein 1-like*	10.87	Protein binding
BAB89348.1	*1-aminocyclopropane-1-carboxylate synthase*	10.67	
KAH9793099.1	*Vascular-related protein 1*	10.60	
LOC127787815	*polygalacturonase-like isoform X2*	10.25	Polygalacturonase activity
LOC127792636	*(S)-N-methylcoclaurine 3′-hydroxylase isozyme 2*	9.97	Oxidoreductase activity, acting on paired donors, with incorporation or reduction of molecular oxygen
LOC127792270	*transcription factor MYB101*	9.47	
LOC127805642	*GABA transporter 1*	9.45	
LOC127810232	*bidirectional sugar transporter N3-like*	9.15	Sugar transmembrane transporter activity
PSR98642.1	*FMN-dependent NADH-azoreductase*	9.07	
LOC127814200	*EG45-like domain containing protein isoform X2*	9.00	
LOC127796202	*7-deoxyloganetic acid glucosyltransferase-like*	8.94	UDP-glycosyltransferase activity
LOC127805921	*NAC domain-containing protein 1-like*	8.88	DNA binding
LOC127813338	*salutaridine reductase-like isoform X2*	8.76	Oxidoreductase activity, acting on the CH-OH group of donors, NAD or NADP as acceptor
LOC127789396	*hexose carrier protein HEX6-like isoform X1*	8.71	Monosaccharide transmembrane transporter activity
LOC127805642	*GABA transporter 1*	8.67	
AOR05828.1	*xyloglucan endotransglycosylase/hydrolase 10*	8.64	
LOC127808774	*alcohol acyltransferase 9*	8.59	Acyltransferase activity, transferring groups other than amino-acyl groups
LOC114318315	*laccase-15-like isoform X1*	8.49	Hydroquinone:oxygen oxidoreductase activity
LOC127795412	*GDSL esterase/lipase 5-like*	8.30	Hydrolase activity, acting on ester bonds
LOC127793042	*myb-related protein 305-like*	8.25	Sequence-specific DNA binding
LOC127790046	*very-long-chain aldehyde decarbonylase CER3-like isoform X1*	8.02	Oxidoreductase activity
PSS24712.1	*Axin-1 like*	7.97	
LOC127790278	*cytochrome P450 98A2*	7.94	Oxidoreductase activity, acting on paired donors, with incorporation or reduction of molecular oxygen
LOC127801145	*trehalose-phosphate phosphatase H isoform X1*	7.89	Trehalose-phosphatase activity
LOC127805724	*protein SMALL AUXIN UPREGULATED RNA 12-like*	7.84	
LOC127807158	*tetrahydroberberine oxidase-like*	7.73	Oxidoreductase activity
AID51423.1	*ethylene response factor 18*	7.64	
LOC122583038	*22.0 kDa heat shock protein*	7.54	
LOC127787260	*acidic endochitinase-like*	7.44	Chitinase activity
LOC127803070	*rhamnogalacturonate lyase B isoform X3*	7.43	Carbohydrate binding
LOC127796343	*AP2/ERF and B3 domain-containing transcription factor RAV1-like*	7.34	DNA-binding transcription factor activity
GFY95051.1	*cupredoxin superfamily protein*	7.08	
LOC127796343	*AP2/ERF and B3 domain-containing transcription factor RAV1-like*	7.01	DNA-binding transcription factor activity
LOC127807942	*monooxygenase 2-like*	6.97	FAD binding
LOC127791424	*glycosyltransferase At3g07620*	6.90	Glycosyltransferase activity
LOC127802236	*proline-rich receptor-like protein kinase PERK7*	6.87	Protein kinase activity
LOC8279466	*cytosolic sulfotransferase 15*	6.75	Sulfotransferase activity
LOC127795644	*23 kDa jasmonate-induced protein-like*	6.72	
LOC127797898	*11-beta-hydroxysteroid dehydrogenase A-like*	6.70	Oxidoreductase activity
LOC127789041	*UDP-glycosyltransferase 73C3-like*	6.69	UDP-glycosyltransferase activity
LOC127797462	*peroxidase 4-like*	6.66	Peroxidase activity
LOC127797581	*cytochrome P450 86A1-like*	6.61	Oxidoreductase activity, acting on paired donors, with incorporation or reduction of molecular oxygen
LOC127805525	*putative polyol transporter 1*	6.60	Carbohydrate:proton symporter activity
LOC127786959	*(+)-neomenthol dehydrogenase-like*	6.59	
LOC127787695	*respiratory burst oxidase homolog protein A-like*	6.58	NAD(P)H oxidase H_2_O_2_-forming activity
LOC127791004	*mitochondrial phosphate carrier protein 3*, *mitochondrial-like*	6.22	Phosphate transmembrane transporter activity
PSS31544.1	*Rhamnogalacturonate lyase*	5.99	
LOC127796209	*galacturonosyltransferase-like 9*	5.90	Glycosyltransferase activity
AOR05829.1	*xyloglucan endotransglycosylase/hydrolase 11*	5.88	
LOC127797011	*classical arabinogalactan protein 5-like*	5.86	
BAB89349.1	*1-aminocyclopropane-1-carboxylate synthase*	5.81	
LOC127796404	*trehalose-phosphate phosphatase 2 isoform X3*	5.79	Trehalose-phosphatase activity
LOC127808654	*adenylate-forming reductase 03009*	5.77	
LOC127809972	*polygalacturonase At1g48100-like*	5.60	Polygalacturonase activity
LOC127807241	*ethylene-responsive transcription factor RAP2-1-like*	5.54	DNA-binding transcription factor activity
LOC127799926	*CASP-like protein 1*	5.54	
LOC127798463	*transcription factor MYB48*	5.48	Sequence-specific DNA binding
LOC127805880	*inositol oxygenase 1-like isoform X2*	5.47	Inositol oxygenase activity
LOC127803057	*elongation of fatty acids protein 3-like*	5.38	Fatty acid elongase activity
LOC127803147	*methyltransferase PMT19 isoform X1*	5.38	Methyltransferase activity
LOC127786954	*(+)-neomenthol dehydrogenase-like*	5.38	Oxidoreductase activity, acting on the CH-OH group of donors, NAD or NADP as acceptor
LOC127797620	*UDP-glucosyltransferase 29-like*	5.38	UDP-glycosyltransferase activity
AGA15800.1	*ethylene response factor 9*	5.27	
LOC127809456	*transcription factor MYB108-like*	5.25	DNA-binding transcription factor activity
LOC127809221	*ethylene-responsive transcription factor ERF008-like*	5.24	DNA-binding transcription factor activity
LOC127811484	*tabersonine 16-hydroxylase 2-like*	5.17	Oxidoreductase activity, acting on paired donors, with incorporation or reduction of molecular oxygen
LOC127813020	*cytochrome P450 711A1 isoform X1*	5.16	Oxidoreductase activity, acting on paired donors, with incorporation or reduction of molecular oxygen
LOC127786644	*cytochrome P450 CYP72A219-like*	5.14	Oxidoreductase activity, acting on paired donors, with incorporation or reduction of molecular oxygen
LOC127792028	*xyloglucan endotransglucosylase protein 1-like*	5.13	Hydrolase activity, hydrolyzing O-glycosyl compounds
LOC127804981	*L-type lectin-domain containing receptor kinase IX.1-like isoform X2*	5.12	ATP binding
LOC127789757	*protein kinase At2g41970*	5.10	Protein tyrosine kinase activity
ANO39898.1	*ethylene response factor 24*	5.10	
LOC127811155	*trans-cinnamate 4-monooxygenase*	5.10	Trans-cinnamate 4-monooxygenase activity
LOC127798547	*UDP-glycosyltransferase 88A1-like*	5.06	UDP-glycosyltransferase activity
EOY16099.1	*Structural constituent of ribosome*, *putative*	5.03	
LOC127808940	*mitogen-activated protein kinase kinase kinase 5-like isoform X1*	4.97	ATP binding
LOC127797621	*beta-D-glucosyl crocetin beta-1*,*6-glucosyltransferase-like isoform X8*	4.95	UDP-glycosyltransferase activity
LOC127806048	*putative GTP diphosphokinase RSH1*, *chloroplastic*	4.85	
LOC127809200	*D-galacturonate reductase-like*	4.81	Aldose reductase (NADPH) activity
LOC127795794	*pleiotropic drug resistance protein 2-like*	4.79	ATP binding
LOC127788535	*ethylene-overproduction protein 1*	4.79	Protein binding
AVR54525.1	*MYB transcription factor*	4.78	
LOC127808842	*cytochrome P450 81Q32-like*	4.72	Oxidoreductase activity, acting on paired donors, with incorporation or reduction of molecular oxygen
LOC127798669	*transcription factor MYB73-like*	4.71	DNA-binding transcription factor activity, RNA polymerase II-specific
LOC127802382	*acyl-CoA-binding domain-containing protein 5-like*	4.60	Protein binding
LOC127801844	*cytokinin riboside 5′-monophosphate phosphoribohydrolase LOG1-like isoform X3*	4.60	Hydrolase activity, hydrolyzing N-glycosyl compounds
PSS05082.1	*Phenylalanine ammonia-lyase*	4.59	
LOC127804247	*protein MIZU-KUSSEI 1-like*	4.53	
LOC114265924	*myb-binding protein 1A-like protein*	4.50	Transcription factor binding
LOC127805731	*UDP-glycosyltransferase 74F2-like*	4.47	UDP-glycosyltransferase activity
AHE13906.1	*xyloglucan endotransglucosylase/hydrolase 9*	4.43	
LOC127788427	*glycosyltransferase family 92 protein RCOM_0530710*	4.32	
LOC127806120	*galactinol—sucrose galactosyltransferase 6*	4.30	
LOC127796158	*glucuronoxylan glucuronosyltransferase IRX7*	4.29	Glycosyltransferase activity
LOC127810184	*transcription factor BHLH089-like*	4.26	DNA-binding transcription factor activity
LOC127792240	*fasciclin-like arabinogalactan protein 9*	4.25	
PSR84714.1	*Galacturonosyltransferase 12*	4.23	
LOC127807192	*beta-galactosidase 16-like*	4.22	Beta-galactosidase activity
LOC127790738	*protein ORANGE-LIKE*, *chloroplastic isoform X1*	4.17	
LOC127802040	*flavin-containing monooxygenase FMO GS-OX5-like isoform X2*	4.16	N,N-dimethylaniline monooxygenase activity
KAJ4723831.1	*Caffeic acid O-methyltransferase*	4.14	
PSS04611.1	*FAD synthase*	4.09	
LOC127802877	*gallate 1-beta-glucosyltransferase 84A24*	4.08	UDP-glycosyltransferase activity
LOC127796482	*LOB domain-containing protein 15*	4.06	
LOC127801122	*axial regulator YABBY 1*	4.04	
PSR93351.1	*Pectinesterase inhibitor domain protein*	3.98	
PSR91886.1	*Chaperone protein like*	3.95	
LOC127800265	*lipase-like isoform X1*	3.94	
LOC127794167	*dammarenediol II synthase-like*	3.94	Beta-amyrin synthase activity
PSS02795.1	*Plant intracellular Ras-group-related LRR protein*	3.92	
LOC127814076	*auxin-responsive protein SAUR36-like*	3.91	
LOC127797571	*ethylene-responsive transcription factor ERF113-like*	3.90	DNA-binding transcription factor activity
LOC127799406	*polygalacturonase*	3.88	Polygalacturonase activity
LOC127807635	*aldehyde oxidase GLOX*	3.83	
LOC127793192	*scarecrow-like protein 27*	3.82	DNA-binding transcription factor activity
LOC127807986	*thioredoxin-like protein CXXS1*	3.76	
LOC127799531	*sugar transporter ERD6-like 16*	3.75	Sugar transmembrane transporter activity
LOC127805879	*zinc finger A20 and AN1 domain-containing stress-associated protein 5-like*	3.75	Zinc ion binding
LOC127793707	*oxygen-evolving enhancer protein 3*, *chloroplastic*	3.72	Electron transporter, transferring electrons within the cyclic electron transport pathway of photosynthesis activity
GFS45640.1	*thioredoxin superfamily protein*	3.70	
BAH89267.1	*putative leucoanthocyanidin reductase*	3.69	
LOC127813769	*cytokinin dehydrogenase 7*	3.69	Cytokinin dehydrogenase activity
AYW01721.1	*persimmon protein ERF25*	3.67	
PSR93233.1	*Acetyl-coenzyme A synthetase*	3.62	
LOC127802856	*(+)-neomenthol dehydrogenase-like*	3.54	Oxidoreductase activity, acting on the CH-OH group of donors, NAD or NADP as acceptor
LOC127802400	*NAC domain-containing protein 104-like*	3.53	DNA binding
LOC127813180	*ethylene-responsive transcription factor ERF061*	3.52	DNA-binding transcription factor activity
LOC127798691	*EIN3-binding F-box protein 1-like*	3.49	Protein binding
LOC127810431	*glucan endo-1*,*3-beta-glucosidase-like*	3.48	Hydrolase activity, hydrolyzing O-glycosyl compounds
LOC127811516	*fructokinase-4*	3.47	Fructokinase activity
LOC127797006	*cyclin-dependent protein kinase inhibitor SMR13-like*	3.45	
LOC127797433	*ATP-dependent 6-phosphofructokinase 4*, *chloroplastic isoform X1*	3.44	6-phosphofructokinase activity
LOC127804052	*beta-D-xylosidase 6*	3.43	Alpha-L-arabinofuranosidase activity
LOC127799002	*putative beta-D-xylosidase*	3.43	Alpha-L-arabinofuranosidase activity
LOC127797597	*pyruvate decarboxylase 1-like isoform X2*	3.42	Carboxy-lyase activity
LOC127790127	*ethylene-responsive transcription factor RAP2-7-like isoform X2*	3.39	DNA-binding transcription factor activity
LOC127809307	*chloroplast protein FOR GROWTH AND FERTILITY 2-like*	3.35	
LOC127808885	*thylakoidal processing peptidase 2*, *chloroplastic isoform X3*	3.29	Serine-type endopeptidase activity
LOC127807957	*protein MULTIPLE CHLOROPLAST DIVISION SITE 1 isoform X2*	3.26	
**Downregulated**			
LOC127809111	*alpha-galactosidase 1-like*	−12.79	Hydrolase activity, hydrolyzing O-glycosyl compounds
AIL29216.1	*fatty acid hydroperoxide lyase*	−8.61	
LOC127803467	*protein CELLULOSE SYNTHASE INTERACTIVE 1*	−8.19	Microtubule binding
LOC127791627	*receptor-like protein kinase At5g24010*	−7.90	Transmembrane receptor protein tyrosine kinase activity
LOC127796215	*2-methylene-furan-3-one reductase*	−7.70	Oxidoreductase activity, acting on the CH-CH group of donors, NAD or NADP as acceptor
KAF3627153.1	*putative caffeoyl-CoA O-methyltransferase 5-like*	−7.63	
GFY98677.1	*stress response NST1-like protein*	−7.57	
PSR89435.1	*Callose synthase*	−7.34	
LOC127790844	*dirigent protein 22-like*	−7.34	
LOC127797089	*nucleobase-ascorbate transporter 12*	−7.28	Transmembrane transporter activity
LOC127813927	*pectin acetylesterase 8-like isoform X1*	−7.16	Pectin acetylesterase activity
LOC127788263	*serine/threonine-protein kinase PBL16 isoform X1*	−6.94	Protein kinase activity
LOC127786732	*monocopper oxidase-like protein SKU5*	−6.91	Copper ion binding
LOC127803982	*allene oxide cyclase*, *chloroplastic-like*	−6.76	Allene-oxide cyclase activity
LOC127808680	*acetyl-CoA carboxylase 1-like*	−6.72	Acetyl-CoA carboxylase activity
LOC127799558	*peroxidase 12-like*	−6.57	Peroxidase activity
LOC127803039	*xyloglucan galactosyltransferase XLT2-like*	−6.56	Galactosyltransferase activity
LOC127789537	*polygalacturonase inhibitor-like*	−6.35	Protein binding
LOC127810198	*cell wall/vacuolar inhibitor of fructosidase 2*	−6.25	Enzyme inhibitor activity
LOC127800689	*LIM domain-containing protein WLIM1*	−6.25	Actin filament binding
LOC127799017	*protein NETWORKED 1A-like*	−6.20	Actin filament binding
LOC127805837	*cellulose synthase-like protein D3*	−6.11	Cellulose synthase (UDP-forming) activity
LOC127801708	*formin-like protein 1*	−5.99	Actin filament binding
LOC127789271	*cellulose synthase-like protein G3*	−5.88	Cellulose synthase (UDP-forming) activity
PSS04488.1	*Trichohyalin like*	−5.82	
GFS34626.1	*thioredoxin M-type 4*	−5.82	
LOC127809708	*lysine-rich arabinogalactan protein 18-like*	−5.81	
LOC127787238	*berberine bridge enzyme-like 6*	−5.80	FAD binding
LOC127801919	*reticulon-like protein B2*	−5.79	
LOC127806788	*leucine-rich repeat extensin-like protein 2*	−5.71	Protein binding
PON73688.1	*Tol-Pal system beta propeller repeat-containing protein*	−5.70	
LOC127796357	*WAT1-related protein At3g28050-like isoform X1*	−5.62	Transmembrane transporter activity
LOC127814265	*acyl-lipid (9-3)-desaturase-like*	−5.61	Oxidoreductase activity, acting on paired donors, with oxidation of a pair of donors resulting in the reduction of molecular oxygen to two molecules of water
LOC127813587	*xyloglucan galactosyltransferase GT19*	−5.59	Glycosyltransferase activity
LOC127814002	*GDSL esterase/lipase At4g01130*	−5.57	Hydrolase activity, acting on ester bonds
PSS26602.1	*Polyketide cyclase*	−5.49	
LOC127795202	*senescence/dehydration-associated protein At4g35985*, *chloroplastic-like isoform X1*	−5.41	
LOC127786623	*scopoletin glucosyltransferase-like*	−5.37	UDP-glycosyltransferase activity
PSS07274.1	*Protein FAM133B like*	−5.33	
LOC127799436	*momilactone A synthase-like*	−5.26	
LOC127804145	*callose synthase 3-like*	−5.24	1,3-beta-D-glucan synthase activity
LOC127800990	*germin-like protein 5-1*	−5.21	Manganese ion binding
LOC127795475	*xyloglucan glycosyltransferase 5*	−5.18	Glycosyltransferase activity
LOC127802055	*glycosyltransferase BC10*	−5.15	Glycosyltransferase activity
LOC127799885	*protein trichome birefringence-like 6*	−5.13	O-acetyltransferase activity
LOC127798217	*pectinesterase/pectinesterase inhibitor 34*	−5.12	Pectinesterase inhibitor activity
PSS21721.1	*ATP phosphoribosyltransferase regulatory subunit like*	−5.11	
LOC127792829	*flavonol 3-O-glucosyltransferase UGT89B1-like*	−5.11	UDP-glycosyltransferase activity
LOC127795273	*trimethyltridecatetraene synthase-like*	−5.03	Oxidoreductase activity, acting on paired donors, with incorporation or reduction of molecular oxygen
LOC127792573	*aconitate hydratase*, *cytoplasmic isoform X2*	−5.02	Aconitate hydratase activity
LOC127810619	*U-box domain-containing protein 6-like*	−4.92	Ubiquitin-protein transferase activity
LOC127788941	*RING-H2 finger protein ATL66-like*	−4.92	
KAG5222891.1	*myotubularin-related protein*	−4.85	
LOC127798906	*protein REDUCED WALL ACETYLATION 2*	−4.83	Acetyltransferase activity
PSS21630.1	*Glucan endo-1*,*3-beta-glucosidase*	−4.63	
LOC114306531	*rootletin-like isoform X3*	−4.61	
LOC127807864	*cellulose synthase A catalytic subunit 2 [UDP-forming]-like isoform X2*	−4.60	Cellulose synthase (UDP-forming) activity
LOC127788688	*tetrapyrrole-binding protein*, *chloroplastic*	−4.52	Tetrapyrrole binding
LOC127810642	*ubiquinol oxidase*, *mitochondrial-like*	−4.50	Alternative oxidase activity
LOC127788265	*mitochondrial uncoupling protein 1*	−4.47	Oxidative phosphorylation uncoupler activity
LOC127808097	*protein LIKE COV 1-like isoform X2*	−4.43	
LOC127793879	*cellulose synthase A catalytic subunit 7 [UDP-forming]*	−4.42	Cellulose synthase (UDP-forming) activity
LOC127791234	*aldehyde oxidase GLOX*	−4.41	
LOC127814001	*rho GDP-dissociation inhibitor 1-like*	−4.40	Rho GDP-dissociation inhibitor activity
LOC127814067	*zerumbone synthase isoform X2*	−4.38	Oxidoreductase activity
LOC127808689	*L-ascorbate oxidase homolog*	−4.32	Oxidoreductase activity
LOC127792648	*polyol transporter 4*	−4.26	Carbohydrate transmembrane transporter activity
GFZ03086.1	*1*,*3-beta-glucan synthase component*	−4.24	
LOC127807082	*putative HVA22-like protein g*	−4.16	
PSS30372.1	*Protein rolling stone like*	−4.15	
LOC127797069	*14 kDa proline-rich protein DC2.15-like*	−4.14	
KAI8014341.1	*Armadillo repeat-containing protein 6*	−4.13	
XP_023882653.1	*protein WVD2-like 7 isoform X1*	−4.02	
LOC127795124	*cytochrome P450 736A117-like*	−3.98	Oxidoreductase activity, acting on paired donors, with incorporation or reduction of molecular oxygen
LOC127795780	*protein trichome birefringence-like 5*	−3.96	O-acetyltransferase activity
LOC127796448	*microtubule-associated protein 70-2*	−3.92	Microtubule binding
LOC127790491	*dirigent protein 22-like*	−3.91	
LOC127803088	*lysine-rich arabinogalactan protein 18*	−3.84	
LOC127789071	*protein WVD2-like 7 isoform X2*	−3.81	
LOC127800498	*linoleate 9S-lipoxygenase 5 isoform X2*	−3.81	Metal ion binding
LOC127791405	*peroxidase 3-like*	−3.79	Peroxidase activity
LOC118056996	*cytochrome P450 71D10-like*	−3.79	Oxidoreductase activity, acting on paired donors, with incorporation or reduction of molecular oxygen
LOC127793976	*jasmonate ZIM domain-containing protein 1-like*	−3.66	
LOC127803184	*WAT1-related protein At4g15540-like*	−3.65	Transmembrane transporter activity
LOC127810929	*fasciclin-like arabinogalactan protein 7*	−3.60	
GFS42279.1	*hydroxyproline-rich glycoprotein family protein*	−3.58	
LOC127787381	*glutathione synthetase*, *chloroplastic*	−3.55	Glutathione synthase activity
PSS33484.1	*hydroxyproline-rich glycoprotein family protein*	−3.53	
LOC127812477	*cinnamoyl-CoA reductase 1-like*	−3.49	Oxidoreductase activity, acting on the CH-OH group of donors, NAD or NADP as acceptor
LOC127795300	*protein trichome birefringence-like 14*	−3.46	Acetyltransferase activity
LOC127800790	*protein WVD2-like 3*	−3.45	Microtubule binding
AOR05817.1	*pectinesterase 3*	−3.43	
LOC127813752	*arabinosyltransferase XEG113 isoform X4*	−3.41	Arabinosyltransferase activity
LOC127797224	*UDP-glucose 6-dehydrogenase 1-like*	−3.32	UDP-glucose 6-dehydrogenase activity
LOC127808089	*alcohol dehydrogenase-like 1*	−3.31	S-(hydroxymethyl)glutathione dehydrogenase [NAD(P)+] activity

## Data Availability

The original contributions presented in this study are included in the article. Further inquiries can be directed to the corresponding authors.
